# Erratum

**DOI:** 10.1167/tvst.9.12.21

**Published:** 2020-11-16

***Erratum in:*** “Assessing the Interocular Symmetry of Foveal Outer Nuclear Layer Thickness in Achromatopsia” by Rebecca R. Mastey, Mina Gaffney, Katie M. Litts, Christopher S. Langlo, Emily J. Patterson, Margaret R. Strampe, Angelos Kalitzeos, Michel Michaelides, and Joseph Carroll (*Trans Vis Sci Tech*. 2019;8(5):21), https://doi.org/10.1167/tvst.8.5.21.

In Figure 4, the 95% confidence intervals (shaded area) for the bias and limits of agreement were calculated in error, using an *n* = 48 instead of an *n* = 76 in the formula specified by Bland and Altman. Figure 4 has been corrected with the proper 95% confidence intervals, which are now slightly narrower than originally published. This correction does not affect the significance of any of the results nor any of the conclusions presented in the manuscript.

